# Epigenetic profiling of MUTYH, KLF6, WNT1 and KLF4 genes in carcinogenesis and tumorigenesis of colorectal cancer

**DOI:** 10.1051/bmdcn/2019090422

**Published:** 2019-11-14

**Authors:** Kosar Babaei, Roya Khaksar, Tahereh Zeinali, Hossein Hemmati, Ahmadreza Bandegi, Pirouz Samidoust, Mohammad Taghi Ashoobi, Hooman Hashemian, Kourosh Delpasand, Fereshteh Talebinasab, Hoora Naebi, Seyed Hossein Mirpour, Arman Keymoradzadeh, Seyedeh Elham Norollahi

**Affiliations:** 1 Department of Biology, Islamic Azad University of Tonekabon Branch Tonekabon Iran; 2 Gastrointestinal and Liver Diseases Research Center, Guilan University of Medical Sciences Rasht Iran; 3 Razi Clinical Research Development Unit, Guilan University of Medical Sciences Rasht Iran; 4 Department of Biochemistry, Faculty of Medicine, Semnan University of Medical Sciences Semnan Iran; 5 Department of Surgery, Poursina Hospital, Guilan University of Medical Sciences Rasht Iran; 6 Pediatric Diseases Research Center,Guilan University of Medical ciences Rasht Iran; 7 School of Medicine, Kurdistan University of Mdical Ciences Sanandaj Iran; 8 Department of Hematology and Oncology, Razi hospital, School of Medicine, Guilan University of Medical Sciences Rasht Iran

**Keywords:** Epigenetics, Fluctuations, Tumorigenesis, Carcinogenesis

## Abstract

Colorectal cancer (CRC) is distinguished by epigenetic elements like DNA methylation, histone modification, histone acetylation and RNA remodeling which is related with genomic instability and tumor initiation. Correspondingly, as a main epigenetic regulation, DNA methylation has an impressive ability in order to be used in CRC targeted therapy. Meaningly, DNA methylation is identified as one of most important epigenetic regulators in gene expression and is considered as a notable potential driver in tumorigenesis and carcinogenesis through gene-silencing of tumor suppressors genes. Abnormal methylation situation, even in the level of promoter regions, does not essentially change the gene expression levels, particularly if the gene was become silenced, leaving the mechanisms of methylation without any response. According to the methylation situation which has a strong eagerness to be highly altered on CpG islands in carcinogenesis and tumorigenesis, considering its epigenetic fluctuations in finding new biomarkers is of great importance. Modifications in DNA methylation pattern and also enrichment of methylated histone signs in the promoter regions of some certain genes like *MUTYH, KLF4/6* and *WNT1* in different signaling pathways could be a notable key contributors to the upregulation of tumor initiation in CRC. These epigenetic alterations could be employed as a practical diagnostic biomarkers for colorectal cancer. In this review, we will be discuss these fluctuations of *MUTYH, KLF4/6* and *WNT1* genes in CRC.

## 1 Introduction

Colorectal cancer is one of the leading causes of death worldwide and is one of the most common malignancies in the digestive tract [1]. More than 600,000 deaths per year occur due to the effects of various types of cancer [2]. Approximately, 98% of colorectal cancers are adenocarcinoma and often occur in adenomatous polyps [3]. The key factor in colorectal cancer is genetic variation (gene mutation) and also epigenetic. In this case, the normal epithelial cells become malignant in colon cells [4]. Often, these changes are caused in oncogene, tumor suppressor and DNA repair genes. Correspondingly, the tumor suppressor genes are silent and the oncogenes that play a key role in the invasion process are activated. Remarkably, Gene mutations can make small or large changes in the genome [5]. Colorectal cancer is seen in forms including: Sporadic form which is the most common type of disease. This disease occurs without hereditary or familial background and usually occurs after 50 years of age. The Adenomatous polyposis coli (APC) gene mutation is seen in the early stages of the formation of a sporadic form of cancer. Here can be mentioned other genes such as the inactivation of the p53 tumor suppressor gene seen in the late stages of adenocarcinoma, which also contributes to the metastasis process. Another form is family one which is about 25% of the patients are in this group and also is considered as hereditary form which is often familial adenomatous polyposis (FAP) and hereditary non-polyposis colorectal cancer (HNPCC).

## 2 Epigenetic and cancer

Epigenetics consist of DNA methylation, histone modification, histone acetylation, histone phosphorylation and also RNA remodeling which DNA methylation is the most important element [6–12]. Meanwhile, alongside with the investigation of epigenetics fluctuation in GI cancers, gene expression study must be added in order to have a better comparison and result [13–20].

**Fig. 1:**
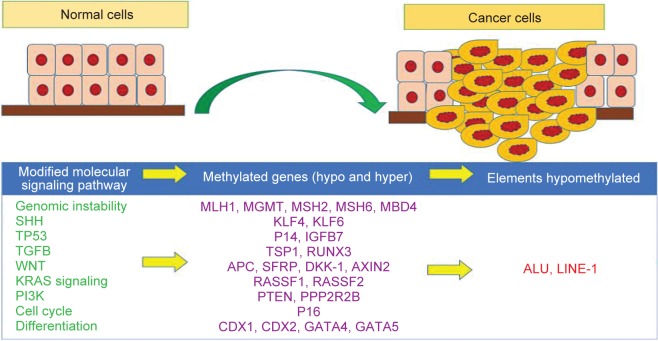
Methylation situation of modified molecular signaling pathways including SHH, WNT, KRAS signaling, PI3K alongside with their important genes.

Remarkably, epigenetic agents play a very important role in carcinogenesis. In this way, besides the genetic factors, epigenetic one is of great importance. Meaningly, epigenetic is a controlled reversible process that causes inherited changes in the expression of genes independent of changes in the nucleotide sequence of DNA [21, 22]. Today, epigenetic mechanisms are known to be a factor in cancer development [23]. Studies have shown that colorectal cancer, like other cancers, is associated with epigenetic changes that make these changes without changing the initial DNA sequence [24, 25], It causes changes in abnormal regulation of transcription factors, followed by changes in cell proliferation, cell survival, as well as cell differentiation (Figure 1) [25–28]. In cells transformed into cancerous cells, epigenetic changes occur at the chromosomal level, including DNA methylation, histone changes, and alterations in function and expression of factors involved in regulating the processes of assembly and nucleosome rearrangement [23, 27, 29, 30]. DNA methylation is one of the three epigenetic layers controlling the expression of genes involved in germ cells and also the specific genes of the tissue [31]. About 3 to 6 percent of the cytosine bases in the DNA of mammals are methylated. This methylation affects the expression of the gene, especially when these dinucleotides are located on the CpG islands. These islands are often located in the promoter regions of the genes [32–34]. DNA methylation is the only epigenetic change directly affecting DNA, which is the result of the transfer of a methyl group of 5 adenosylmethionine to C5 of Cytosine base. In mammals, cytosine methylation occurs in the CpG position of the DNA sequence. CpG islands, which are rich in CpG dinucleotides, are a hallmark of the promoter regions of the genes [34]. The complexity of setting DNA methylation patterns is revealed through the diverse function of DNA methyltransferase (DNMTs) and reactive proteins with 5-methyl cytosine. Changes in the pattern of methylation can lead to tumorgenesis and also carcinogenesis. These changes are divided into two categories of hypomethylation and hypermethylation [35]. Methylation occurs through a stable change on the DNA molecule, and mainly on certain CpG islands, in association with the promoter of tumor suppressor genes, while mutations often appear in distinct tumors in different places in a particular gene. Importantly, most CpG islands within the promoter of genes are in nonmethyl normal tissues [36]. Genetically, tumorgenesis in CRC is divided into a multistage process involving mutation activation in protooncogenes and loss of function of tumor suppressor genes. In contrast, DNA repair and recovery genes play an important role in anti-tumor activity by maintaining the integrity of the genome. Conclusively, because of the different mechanism of different genes in different pathways, we selected MUTYH, KLF6, KLF4 and WNT1genes in order to discuss their importance in carcinogenesis and also tumorigenesis in CRC. Intrestingly, all these mentioned genes have a noticeable impression in cancer progression.

## 3 Involvement of certain genes

**Fig. 2:**
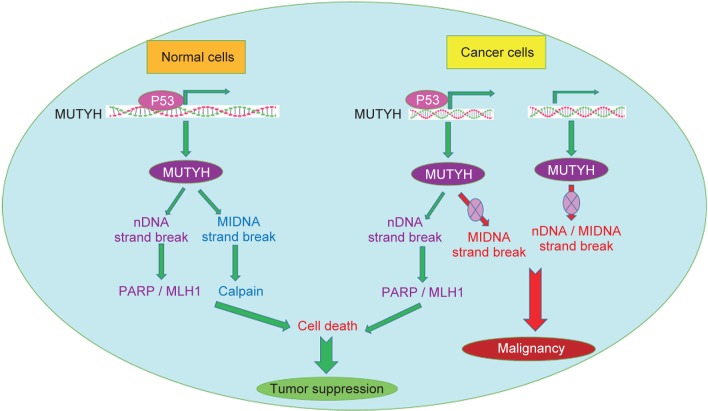
Role of MUTYH gene in normal and cancer cell and also it,s molecular mechanism in both normal and malignant processes.

**Fig. 3:**
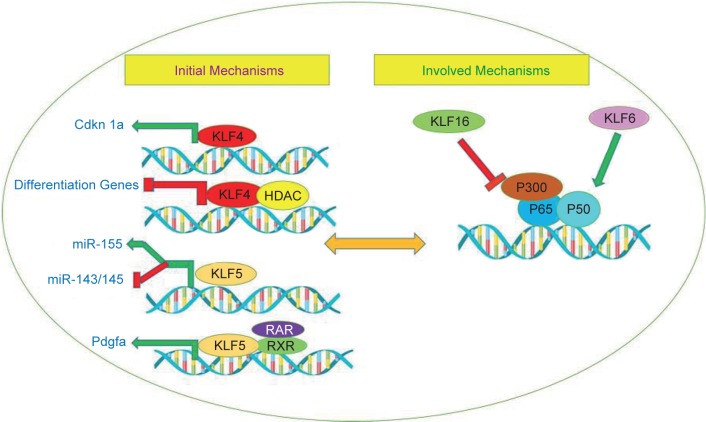
A schematic mechanism of KLFs family genes.

**Fig. 4:**
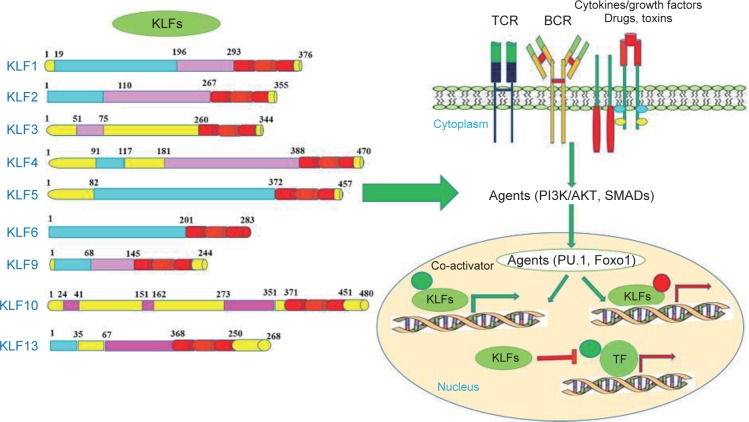
KLFs family gene like KLF4/5/6 and 16 in initial mechanims along side with their involvement in carcinogenesis.

Many genes are involved in different molecular mechanisms in the carcinogenic pathway, including the MUTYH, KLF6, KLF4 and WNT1. Base Excision Repair (BER) is a DNA repair pathway that eliminates mutations and modifies the genome mainly through successive responses, including the diagnosis and processing of damaged nucleotides [37]. The interruption of BER leads to cancer through accumulation of oxidative DNA damage caused that has been observed in MUTYH-related polyposis (MAP). MAP is a hereditary colorectal cancer syndrome that is caused by adenoma and cancers with a cumulative 8-Oxo guanine (8-OxoG), which causes mutation in the mutY homolog E. coli (MUTYH) gene (Figure 2) [38]. The MUTYH gene is located on the short arm of chromosome 1, and its map is P34.3_P32.11 and is about 2.11 kilobases long and consists of 16 exons. This gene encodes a protein containing 535 amino acids, which is a kind of glycosylase. This protein, which is one of the components involved in the recovery of DNA through BER, Was identified in humans in 1995 and contributes to the repair of oxidation damage [39]. During DNA replication, A is paired with T and C with G. During aerobic metabolism of normal cells or in a position that increases the production of ROS, guanine oxidation results in the formation of 8-oxoG which is rapidly and incorrectly coupled to adenosine instead of cytosine. MUTYH Glycosylase removes adenine bases that are weakly coupled with 8-oxoG. This enzyme corrects this error, so increasing these mutations by G: C> T: A would interfere with the control of the cell cycle, leading to the formation of a tumor. The importance of MUTYH gene mutation in colorectal cancer was confirmed by AL-Tassan *et al.* for the first time, people from a family who have two-allelic mutation in the MUTYH gene have a autosomal form of adenomatous familial polyposis,when analyzing the cancerous tissues of these patients, there was a strong indication of the conversion of G: C> T: A into commonly mutated genes of CRC (APC, K-RAS). Later on, other authors emphasized that these findings show MUTYH gene mutation that reduces the effect of the BER system and the emergence of CRC [40–42]. The level of MUTYH gene expression is related to tumor location, tumor size, degree of cell differentiation and depth of invasion to the intestinal wall, angiolymphatic infiltration, and lymph node involvement. In fact, MUTYH is less pronounced in large tumors with invasive lymphatic vessels (malignant stages of colorectal cancer) [43]. In the Western countries, MUTYH is the second leading cause of APC variants in patients with polyposis. Although few studies have been conducted on the study of MUTYH variants in Asian polyposis patients [44]. Changes in disease progression may also be due to accumulation of epithelial mutations or MUTYH hypermethylation [45]. In fact, hypermethylation of the promoter region of the genes involved in the production of BER system is found in a variety of tumors of thyroid, bladder, ovarian, brain, CRC [46]. In molecular genetics, the Krüppel-like family of transcription factors (KLFs) are a set of zinc finger DNA-binding proteins that regulate gene expression. One of the members of this family is the Krüppel-like factor 6 gene (KLF6), Changing the expression of this tumor suppressor gene plays a role in the incidence of cancer. KLF6 is located on the chromosome (10p15). The mutation occurrence in this gene is involved in the formation of some types of tumors, so that mutation in the gene can be related to cancers such as colorectal [47], malignant glioma [48], nasopharyngeal [49], breast [50], Stomach and Prostate Cancer [51, 52]. The KLF family is extensively involved in signal transduction dependent on growth, cell proliferation, development, apoptosis and angiogenesis (Figure 3). In normal cells, KLF6 increases the P21 inhibitor in an independent TP53 type and inhibits growth. It actually breaks the function of kinase dependent on cyclin D1 and causes the cell cycle to stop in the G1 stage. Furthermore, KLF6 acts as an inhibitor of cell proliferation against C-Jun oncoprotein [53, 54]. Down-regulation of KLF6 may contribute to the development of solid human cancers, and inactivating it may result in the development of colorectal cancer as a primary or common occurrence. In general, the inactivation of tumor suppressor genes with their lack of expression results from genetic or epigenetic changes such as mutation, loss of allele, or hypermethylation of the promoter region of the gene [53]. Notably, KLF6 is proposed to be a methylation gene in esophageal squamous cell carcinoma [55]. Correspondingly, KLF4 is another member of the family of transcription factors that regulate various cellular functions, such as growth, development, cell proliferation, differentiation, apoptosis and transcription. The gene is located on chromosome 9 at position q31.29, and the length of this gene is 5.6 kB. The gene contains 5 exons, and the protein encoded by this gene is 470 amino acids. As a transcription factor, binds the CACCC-rich or GC-rich region in the promoter the genes that involved in many cellular processes and suppresses or stimulates the expression of the desired genes. The classification of transcription factors is mainly based on the DNA-binding motif, which contains a highly protected zinc finger motif in its structure [56, 57]. The gene belongs to the SP/KLFs family, of which 17 have been identified in humans (Figure 4) [58]. KLF4 is expressed naturally in the lungs, testicles, skin, thymus, vascular endothelial cells, epithelial cells of the skin, the eyes, the kidneys, the bone and the digestive tract, including the colon [56]. KLF4 is considered to be genes that have dual performance, due to the presence of different domains in this structure. t can act as a tumor suppressor and also an oncogene, which also depends on the type of tumor, tissue or tumor stage, that These changes are done with the help of molecules such as p53 and p21 and p27 through changes in processing or post-translational modifications [58]. This gene plays its role by inhibiting the cell cycle or by anti-apoptotic effect. The expression and activity of KLF4 in human cancers are different. Genetic changes in the gene in cancer are also unusual [59]. Basically, KLF4 in the intestine plays several important roles in the regulation of hemostasis. As, it has a significant effect on the development and final differentiation of Goblet cells involved in mucosal secretion. However, subsequent studies on mice showed that inhibition of this gene prevented the differentiation of enterocytes in the intestine, which also inhibited the expression of carbonic anhydrase in the colon. So in the intestine, KLF4 involves in postbirth maturity, differentiation and proliferation, migration and placement of intestinal epithelial cells, as well as in maintaining the hemostasis of intestinal cells [58–60]. Now, in the case of cancer, there is evidence that promoter hypermethylation and deletion mutation in KLF4 gene can reduce or eliminate the expression of this gene [60]. The KLF4 decreases in cancers such as colon and esophagus, the brain, kidneys, prostate, bladder, and leukemia in advanced stage of the disease. KLF4 has been proven to be one of the few genes that have been reported to be at the onset of Gastrointestinal cancer [56, 59]. KLF is associated with the transmembrane protein E-cadherin, and if KLF4 is not expressed, cellular tissue passes through the epithelial stage to mesenchymal development and invasion of the disease [60, 61]. Conversely, KLF4 increases in breast, nasal Head and neck cancer [59, 60].

**Fig. 5:**
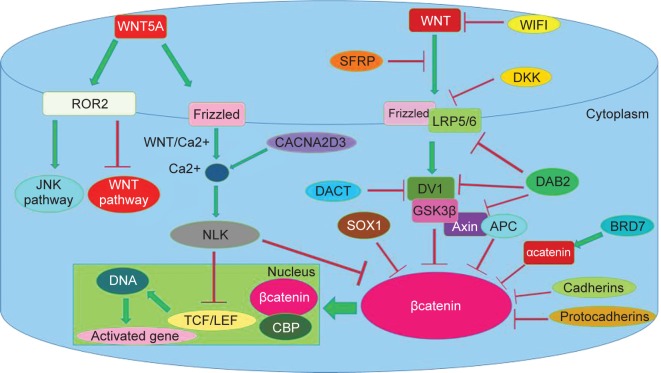
Role of WNTs family with its subclasses genes.

Epigenetic changes, including hypermethylation of the promoter of the KLF4 gene in the colon, reduce the expression of this gene and induction of tumorigenicity [56]. When cells are exposed to gamma rays and free radicals, various mechanisms, including effects on cell cycle, induction of apoptosis, as well as DNA repair processes, are activated [60, 62]. KLF4 is an important regulator for the cell cycle, and cells with excessive expression of KLF4 inhibit the cycle at the G1 / S stage. The cell cycle is controlled by cyclin kinases, the activity of these kinases being regulated by activators or cyclins and inhibitors (CKIs). The KLF4 protein activates inhibitors such as P21, p27 and prevents DNA synthesis. Expression of inhibitors such as p21, which inhibits the cell cycle at the G1 / S stage, is followed by DNA damage. It has been shown that P21-dependent expression of p53 and inhibition of cell cycle is mediated through KLF4 [60]. In cases where the expression of the KLF4 gene is inhibited, such as hypermethylation, other signaling pathways such as WNT that contribute to the intestinal stem cell self-renewing. The KLF4 is also associated to cateninβ and thus effects itself. Both in the familial heredity and sporadic, there is a decrease or no expression of KLF4, and the cancer progresses from the first stage to the fourth stage. The expression level of KLF4 has an inverse relationship with the size of the tumor [56]. These studies have been conducted in countries such as South Africa [56], United States [63], Japan [61], Europe [64], and concluded that the expression of this gene in colon cancer has decreased. In this way, another responsibility that contributes to cancer, is WNT signaling pathways. The mutation in the gene that changes its products is one of the most prominent cases in the development of tumors, and the WNT signaling pathway has not been the exception (Figure 5) [31, 32]. Activating this pathway is important not only at the onset of the carcinogenesis of colorectal cancer but may also control the malignancy potential of the cells in more advanced stages and may be an objective to develop new therapies for this type of cancer. These cells originate from stem cells that are located at the base of the crypt and migrate to the luminal surface of the cavity [65]. Cells that result from the splitting of primary stem cells or as stem cells remain or are differentiated, which is controlled by the WNT signaling pathway. The WNT signaling pathway is a highly regulated pathway from the sponge-to-human species that plays a role in the proliferation, differentiation, and determining the fate of the cell. The WNTs family is part of the glycoproteins and has 19 known members [66–68]. WNT genes secrete cysteine-rich proteins that apply their biological functions through the autocrine or paracrine system [65, 67]. These proteins have between 23-24 subunits of cysteine. This pathway is involved in maintaining hemostasis in many tissues including the digestive tract, skin, bone, hematopoietic system, hair follicle, muscle, liver and brain [66]. During the studies, WNT proteins activate 3 pathways: 1.canonical pathway 2. The planer cell polarity pathway 3.WNT/Ca pathway. Which canonical pathway is importance to us. Increasing the activity of the WNT signaling pathway has been seen in many malignancies, including colon cancer, liver and breast cancer. WNT proteins are produced by mesenchymal fibroblast cells located near the basal lamina [65]. On the membrane of epithelial crypt cells, there is a receptor called Frrizled (Fz), a protein that passes through the membrane several times and is part of the G group of proteins. The amine portion of this receptor is located outside the cell, which is rich in cysteine. WNT connects to this region, this domain is called CRD. Next to the Fz, there is another receptor assistant called the low-density lipoprotein receptorrelated protein 5/6 (LRP5/6) that plays a role in this signaling path. The LRP5/6 is secreted through a chaperone called MESD. By secretion of WNT, the extracellular part of the receptors Fz and LRP5/6 are attached. By conformational change of the Fz receptor, it induces protein phosphorylation called Dishevelled (DVL), which acts as a mediator for signal transmission from the receptors. A number of kinases are known to phosphoryl Dvl, including casein kinase I, casein kinase II. Dvl contributes to phosphorylation of the cytoplasmic portion of LRP5/6, which consists of five protected PPP (S/T) P motifs, which are characterized by Casein kinase 1(CK Iα) and Glycogen synthase kinase 3 β (GSK3β). By phosphorylation of the receptor, Axin drops the LRP and prevents the formation of a destructive complex to destroy the cytoplasmic and important protein called βcatenin (35). βcatenin is known as a transcription factor that is encoded by the CTNNB1 gene and is known as an oncogene in colon and liver cancer, and activates the genes involved in the cancer [65, 69]. β-Catenin binds inside the cell and binds to the T-cell factor / lymphoid enhancer binding factor1 (TCF/LEF1) in the nucleus, which induces expression of target genes [68]. These genes include C-Myc, Cyclin D1, Axin-2, Sox-2, TCF-1 [65]. Now, in the absence of WNT, Axin, CKI, GSK3β and APC form a complex and destroy the β-catenin. For destruction, it is necessary that the β-catenin is phosphorylated in the amino portion by GSK3β and Ck Iα, which Beta-transducin repeats-containing proteins (β-TrCP) serve as the substrate recognition subunits for the E3 ubiquitin ligases. These ligases ubiquitinate specifically phosphorylated substrates and actually labeled and identified and destroyed by protease [68]. WNT1 gene was first discovered by Nusse and varmus in 1982, in mouse that was essentially called Int-1 [65]. This gene is located on the chromosome q13.1212 [70]. WNT1 stimulates the pathway of Canonical WNT / βcatenin, which causes changes in cellular fate or cell formation [71]. WNT1 acts on target genes *via* the TCF / LEF-1 complex. Recent studies have shown that the increased expression of WNT1 has occurred in many cancers, including breast, colon lung and prostate cancers, which, through inhibition of antagonists, have an oncogene role. One of the target genes is C-Myc, which plays a role in proliferation and cell growth, and increases cell cycle transitions from G1 to S by activating the Cyclin E/ cdk2 complex [72]. Besides, the above explanation of molecular signaling pathways, for these genes in treatment category, Gut microbiota is recommended [73].

## 4 Conclusions

Studies have shown changes in the expression of MUTYH, KLF6, KLF4 and WNT1 genes in various cancers. On the other hand, environmental factors play an important role in epigenetic genes (including DNA methylation), and this change in methylation causes changes in the expression of these genes. Correspondingly, studies indicate that these genes have the diagnostic potentially and the study of the expression of these genes that contribute to the incidence of cancer as well as the determination of their methylation in different populations can help determine the molecular causes of CRC in order to employ them a s a main prognostic biomarkers in cancer detection [74].
